# Designing an optimal model of blood logistics management with the possibility of return in the three-level blood transfusion network

**DOI:** 10.1186/s12913-023-10240-0

**Published:** 2023-11-27

**Authors:** Monireh Ahmadimanesh, Hamid Reza Safabakhsh, Sedigheh Sadeghi

**Affiliations:** 1 Department of Industrial Engineering, Faculty of Industry and Mechanic, Sajjad University, Mashhad, Iran; 2 Blood Research Center, Higher Institute of Blood Transfusion Education and Mashhad Regional Blood Transfusion Training Center, Mashhad, Iran; 3 Faculty of Mathematical Sciences, Ferdowsi University of Mashhad, Mashhad, Iran

**Keywords:** Blood logistics management, Three-level blood transfusion network, Simulation, Neural network

## Abstract

**Background:**

Inventory managers in the blood supply chain always endeavor to provide their clients with prompt and appropriate responses. On the other hand, timely and regular blood deliveries to consumers are essential since ineffective delivery and transportation practices promote shortages, returns, blood loss. The paper attempted to develop an extensive and integrated optimal model of blood transfusion network logistics management by blood type to reduce the cost of losses, returns, and blood shortages given the relevance of this for the blood transfusion network.

**Methods:**

The regional blood transfusion network in Khorasan Razavi, which contains one main base, six central bases, and 54 hospitals, should be constructed using the optimal model for blood inventory management and distribution. A reusable simulation process was used to identify the optimal behavior for the inventory of all participants in the region (central bases as suppliers and hospitals as consumers), and the demand of hospitals as consumers has been calculated using artificial neural networks. This will lead to a significant reduction of returned blood units by consumers, optimal management of suppliers’ and consumers’ inventory to prevent waste and shortages. The routing method was used to proceed with the designed model and look into the optimal strategy to distribute blood requested by the consumers. with the aim of reducing the cost and increasing the speed of transportation.

**Results:**

The model’s solution allowed for the estimation of the amount of consumers’ demand, the optimal amount of target stock, the central bases and hospitals’ reorder points, as well as the method of distributing blood from the supplier to its consumers. Implementing the model leads to outcomes such as reducing the time of blood transfer from the central bases to their consumers, increasing the speed of blood delivery to the consumers, increasing the average stock of blood in the central bases, reducing the accumulation of distribution machines at the location of the central bases, the amount of stock, the method for requesting, consuming, and storing blood, and the performance of the central bases’ consumers all affect how much control they have over them.

## Background

### Introduction

Three levels can be used to describe blood bank inventory management: the hospital level (Hospital Blood Bank), the regional level (Regional Blood Bank), and the overall blood transfusion organization. As a result of the many bases and hospitals in regional systems, the numerous ongoing operations, and the complexity of the organizational structure, RBB inventory management will be more important than HBB management among these levels [1, 2]. Because the regional blood center director, who creates hospital distribution policies and supervises issues including regional blood collection, distribution, and transfusion planning, is the decision-maker [3].

Iran, a country with a population of about 75 million, is one of the most populous countries in Central Asia. Due to the necessary investment by the Iranian government on the health care system in recent decades, the country has now benefited from the advanced health system infrastructure. Blood transfusion is one of the essential components of the healthcare system that saves the lives of millions of people in the world [4]. On the other hand, according to the statistics provided by the Blood Transfusion Organization, the amount of blood waste in the country is over 12%. About 14% of this waste is associated with contaminated and viral blood, and 60% of it results from hospitals and blood banks having less supervision. Blood wastes associated with previous or returned blood account for 9% of this total, compared to 4% in industrialized countries. One of the factors contributing to blood loss is also the ineffective distribution of blood. Therefore, it can be said that this percentage can be reduced by right management and scientific use of blood in hospitals and blood banks [5]. Therefore, the field of blood transfusion requires special policy making and precise planning, which requires conducting applied research to provide clear strategies and develop technology in this field. The blood transfusion organization in Khorasan province, which was established on October 22, 1978, was rated third in terms of output, after Tehran and Fars provinces. With over 450 special patients in the province (each patient requires an average of one to two injections per month, and 10% of the province’s blood is consumed by donors), the presence of specialized and super-specialized treatment facilities, the importance of the holy city of Mashhad in the east of the country, pilgrimage and tourism trips to the province, and the rise in accidents and incidents, it is twice as important to pay attention to inventory management.

In the current state of the studied network. the estimate of the precise daily need of hospitals does not correspond with their real demand since the consumption of blood products in hospitals is a function of the number of daily incidents and accidents and has an unpredictable nature. Hospitals prefer to purchase extra blood products in these circumstances to have a safe supply to deal with any shortages, but doing so creates a significant problem due to the restricted supply of blood available at the local blood center or blood transfusion bases. Another reason that makes ordering more than what hospitals need is problematic is the perishability of blood products. If the blood products ordered by the hospitals are not used before the end of their useful life, they are expired and must be destroyed. Since the costs of obtaining blood products are very high, the expiration of each unit of blood will result in high costs [6]. On the other hand, in the system under study, there is no organized system of distribution, and each consumer, as needed, sends his truck to the appropriate central base to receive the blood he needs, resulting in an unplanned and occasionally delayed delivery as well as a one-time gathering of consumers in the central base. Regarding this, the Blood Transfusion Organization must work to make it possible for all patients nationwide to have access to blood and its products, all the while keeping a close eye on the region’s blood reserves on a daily, monthly, and annual basis and the functioning of the blood supply network [5].

Despite the significance and sensitivity of regional blood inventory management, modeling work in this area frequently focuses on single supplier/consumer systems, and there are not many real-world studies in this field because of the complexity of regional blood inventory management. Additionally, only a small number of studies have addressed regional blood inventory management using reusable modeling (applicable to all bases and blood banks in the region), and none of these studies have made any claims regarding the implementation of their suggested model in real and large scales. Additionally, routing has not been addressed simultaneously in any of the studies that are currently accessible in this subject, and no solution has been offered for delivering the collected blood to customers using machines with various capacities. In this study, an effort is made to design, build, and test an inventory management and distribution solution for regional central blood centers using integrated modeling, using Khorasan Razavi blood transfusion centers as an example. Therefore, in this paper, we attempted to address the following:

- Development and implementation of a general and integrated model of inventory management and distribution in a three-level regional blood transfusion network (regional blood center or main base, community blood centers, hospital).
- Simultaneous implementation of blood inventory management and distribution by blood group and consumer in a wide regional and three-level network.
- Designing the best logistic model possible that can be applied to all participants (hospitals and central bases) in the regional blood transfusion network.

It should be noted that the study provided appropriate and scientific tools and techniques for designing an inventory management model and red cell distribution by the four main blood groups (A, B, O, and AB) in an RBB. Moreover, attempts have been made to formulate the optimal inventory management and distribution policies. Red blood cells are among the components of blood that are in the highest demand and consumption. After conducting this research, it is anticipated that the supply chain and logistics of the studied blood will be improved by identifying appropriate policies in the area of inventory management and distribution with a focus on reducing waste and shortages of blood as well as awareness of the level of demand. Therefore, this research answers the following questions:

1. What is the optimal model of blood logistics management with the possibility of returning goods in the three-level blood transfusion network?
2. What is the optimal level of blood stock in central blood transfusion centers and hospitals addressed in this study?
3. What are the appropriate and optimal routes of blood transfusion to the studied consumers and what is the workload of the blood distribution machines?

In the following the article is organized as follows. The second section looks at the theoretical underpinnings and an overview of the research in the area under question. The proposed model and its relationships are presented in the third section The study findings are given in the fourth section, the model validation method is detailed in the fifth section, the discussion and conclusions are addressed in the sixth section, and suggestions for further research are discussed in the seventh section.

## Literature review

The creation of effective models and procedures for managing the stock of perishable goods has largely been a result of the work of supply chain researchers [7]. From 1970 to 1980, a large portion of the literature review was developed. Jennings [8] offered the first conceptual framework for classifying the blood inventory issue. This initial work examines the issue via three levels of hierarchy (strategic, technical, and operational) and illustrates the impact of implementing different blood supply policies.

A regional blood center in the UK was used to conduct [9] analysis of the inventory management procedures of the blood supply chain. Integer programming models were created by [10] to determine which hospitals a vendor should visit each day (through blood center vehicles). Dynamic random scheduling and simulation were used by [11] to describe the inventory management model of the Dutch Regional Blood Ban. A simulation model that matches the operational characteristics of the blood center inventory system was given by [12]. He has offered suggestions for inventory management by putting this model into practice based on historical data from the Regional Blood Bank of Jiangsu Province from 2005 to 2008. Using the multivariate optimization technique, [13] developed a public network optimization model for the intricate human blood supply chain. They have attempted to reduce expenses associated with shortage and waste by developing a regional blood bank system that consists of distribution centers, testing, processing, storage, and collection bases. The ideal values for each cost were established by solving this model using numerical examples. Baesler et al. [14] examined and proposed inventory policies for the RBB using a discrete event model. The supply chain operations, including blood collection, testing, production, and inventory management, are depicted in the model. Blake and Hardy [3] designed and evaluated regional blood network inventory policies in Canada using a reusable simulation model. They used the level of response technique and nonlinear planning to establish the best possible inventory policies / supplier. For the logistics of blood banks [15] suggested a vehicle routing model. In their paper, they discussed the subject of vehicle routing with delivery and collection in relation to the distribution and collection of blood for a general medical system. To this end, they employed a heuristic model a combination of genetic algorithm and simulation The Icelandic Blood Bank, which contains a distribution center and a processing center, was the location of a research conducted by [16]. The optimal ordering policy to improve the blood supply chain and reduce waste was determined by the study using discrete simulation. Additionally, linear regression analysis was utilized to demonstrate how the blood supply chain might be influenced in order to comprehend it better. The optimal reserve level model’s results reveal the inventory fluctuations and shortage throughout a certain time period. A workable solution method for the management of red blood cells in the treatment center in the regional network was put forward by [17]. To reduce costs in the inventory management system for perishable goods and lower blood center losses, this method was developed and combined with dynamic planning. The optimal ordering and storage policy was discovered by using numerical examples to solve the given model. Artificial neural network (ANN) software was utilized by [18] to predict blood demand in blood transfusion centers. They predicted the monthly need for red blood cells, plasma, and platelets in an RBB, three blood components. Ahmadimanesh et al. [19] developed an optimal inventory management model in a region without blood unit separation using reusable simulation and neural networks. The findings showed a considerable decrease in lesions and a deficiency in blood units. According [20], the problem of blood routing under uncertainty has certain characteristics that distinguish from traditional routing. They suggested a hybrid linear integer to address the blood routing problem in order to describe the programming. Using two models of mixed integer programming and probabilistic robust planning, [21] investigated the issue of blood routing from blood centers to some hospitals under uncertain conditions. To solve these models, data from an actual supply chain was employed. A routing inventory model for perishable goods with high consumption rates was proposed by [22]. The production per day, the sales centers that should be visited each day, the route taken by visitors to the sales centers, and the goods supplied to the sales centers every visit were all factors considered in this model. A numerical example containing a manufacturing node and three sales centers was used to solve the suggested model. The objective function had a value of 15,729.88, according to the answer. According [23], a Monte-Carlo simulation approach is used to model the blood supply chain (BSC) under both supply and demand uncertainty, two different blood supply strategies are generated to analyze the BSC performance. The data used in the validation and verification of the model is a real case study that increases the practical application of the proposed model. The results show that a slight increase in collected blood units will lead to a noticeable increase in minimum and average availability percentage accompanied by an acceptable increase in the total BSC cost, taking into consideration the effect of cost parameters on total cost behavior. Mansur et al. [24] proposes a new mixed-integer linear programming (MILP) model that considers multiple echelons, multiple blood types, transportation and production emissions, and blood bag shelf life to maximize profit in the blood supply chain system. The model is applied to a real-world case study. The research results confirmed that the model could mitigate the BSC challenges. We also conducted sensitivity analysis of patient demand parameters, blood production capacity, selling price, and shelf life to investigate the impact of the key factors affecting the total profit, costs, carbon emissions, and number of obsolete products. Shih et al. [25] formulate a multi-criteria decision-making (MCDM) model for platelet inventory management along the blood supply chain that minimizes three conflicting measures: total supply chain costs, unit outdated, and unit shortage under demand uncertainty. The developed model is solved using three solution techniques: Preemptive goal programming (PGP), non-preemptive goal programming (NPGP), and weighted objective method (WOM). The results indicate that the desired goal for expected supply chain cost is achieved using the PGP model; however, the unit shortage and outdated exceeded by over 350% for each criterion.

In Table 1, a summary of previous studies in the two areas of blood supply chain and logistics and inventory management and transportation of the regional blood bank is discussed.

**Table 1:** A summary of studies conducted on supply chain and blood logistics and inventory management and transportation of the regional blood bank

**The difference with the proposed research model**				**The implementation of the Model**	**Method**	**Purpose**	**The examined stage of the chain**			**Authors**
**Model implementation dimensions**	**method**	**purpose**	**The stage under review**				**distribution**	**inventory**	**collection**	
		☑	☑	American Blood Transfusion Organization	Dynamic planning	Designing ordering policies		☑		[26]
			☑	Blood bank of Turkey	Simulation	Examining the blood bank inventory management policies		☑		[27]
☑	☑		☑	Solving the numerical example	Integer programming Proximity search	Routing	☑			[28]
☑	☑		☑	Testing the designed model using data from the Stanford Blood Services Center	LIFO Analysis FIFO Analysis	Improving inventory management policies and reducing the amount of expired items		☑	☑	[29]
☑			☑	Collecting bases in urban and rural environments in French cities	Simulation	Developing blood collection Scenarios			☑	[30]
☑			☑	Testing the model with real data from several hospitals	Simulation	Reducing blood expiration rate and optimizing ordering policies		☑		[31]
			☑	National Blood Center of Iran	Simulation Taguchi	Improving the efficiency of the blood supply chain		☑		[32]
☑	☑		☑	Solving the numerical example	Integrated integer linear programming	Increasing the efficiency of the supply chain		☑		[33]
☑			☑	Implementing the model in Saskatchewan, Canada	Simulation	Designing a blood distribution network model	☑			[34]
☑	☑		☑	Implementing the model in a hospital	Two-stage stochastic programming	Reducing the costs of expiration, waste and shortage		☑		[35]
☑	☑		☑	Solving the numerical example	Possible optimization	Increasing chain efficiency		☑		[36]
☑			☑	A distribution network with 8 customers and 3 vehicles	Branch and cut	Blood routing	☑			[20]
	☑		☑	Sari blood transfusion network	Mixed integer programming - Robust probabilistic planning method	Blood routing	☑			[21]
☑	☑		☑	Special region in Italy	Binary programming	Organizing blood management system		☑		[37]

A review of previous studies reveals several research. Although there is a significant literature on blood inventory management and distribution very few studies have looked into intricate regional blood transfusion networks. Additionally, the majority of studies on RBB have provided a model that is either realized in a small blood bank or solved using just numerical examples and without actual implementation. In the end, no comprehensive and integrated model of inventory management and distribution in the area has been developed in the research under consideration. The present study differs from previous ones in blood supply chain and logistics because it develops an optimal model of inventory management and blood distribution in the regional network. First, the optimal model of inventory management desined with the aid of simulation and neural network techniques is comprehensive and general and used for all bases (suppliers) on the network; no supplier in the area is required to implement an optimal model of inventory management to re-modeling and spend additional money and time; only with the update of events can be determined. Additionally, in this part of the model, the optimal quantity of the target inventory and reorder point is established for all suppliers and customers in the region. In other words, this approach will considerably aid the provider in predicting the amount of demand and the units sent, but it will also improve consumer ordering habits. Secondly, implementation of this model results in the optimization of the distribution of blood units to the desired hospitals with the least losses, decreasing the time of blood transfer to consumption, increasing delivery speeds, and decreasing the accumulation of distribution machines at supplier locations. The second point is that the multi-objective programming routing model is associated with blood inventory management variables.

## Methods

### Description of the model

**Fig. 1:**
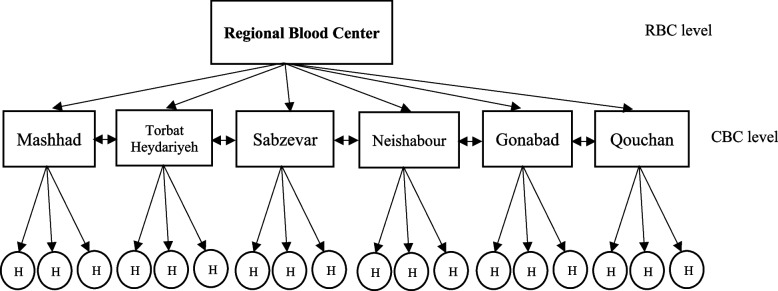
Regional blood structure of Khorasan Razavi’s blood transfusion network

This study aims to optimize the inventory and distribution in the blood logistics chain in Khorasan Razavi blood transfusion organization. The region has three levels of Regional blood center (RBC), Community blood center (CBC) and hospitals as shown in Fig. 1. According to this data, Khorasan Razavi province has 54 hospitals, six CBC located in Mashhad, Sabzevar, Torbat Heydariyeh, Qouchan, Neishabour, and Gonabad, as well as a RBC in the province’s center. Each CBC receives the blood that has been gathered from the mobile and donating bases, tests it as needed, separates the blood components, and then stores and packages it for distribution to its consumers as necessary. RBC in Mashhad supervises the operations of the central bases and coordinates and organizes them. The developed model was implemented using daily data from Khorasan Razavi’s blood transfusion databases between September 2019 and September 2021. The data used in the study were not biological or human samples and were only extract from the archievied data (without the patient’s name) of Razavi’s blood transfusion database.

**Fig. 2:**
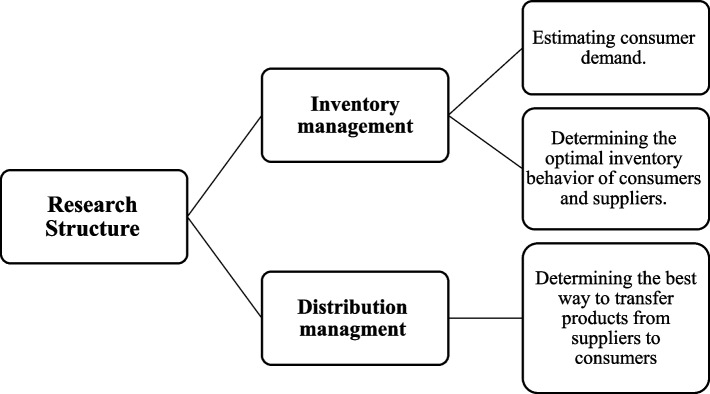
Content structure of logistics management of the present research

Two key components of inventory management and distribution have been taken into consideration in order to optimize blood logistics management in the studied network. The subject of estimating consumer demand, selecting the most appropriate target inventory level, and the reordering point of customers and suppliers have all been covered in the inventory management part. Additionally, in the distribution management, the optimal route with the lowest cost to deliver blood products to consumers is identified by constructing the routing model. As a result, the desired model for implementation in the Razavi Khorasan province’s blood transfusion network (the study’s case) has been completed in three stages: examining the current and desired behavior of consumers, estimating their demand, and designing a routing model. The logistics management content structure that was examined in this study is shown in Fig. 2.

### Research algorithm

**Fig. 3:**
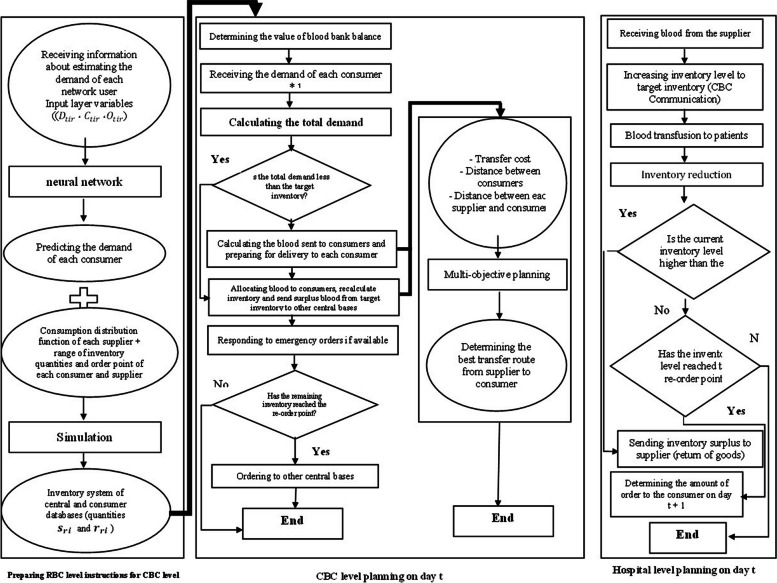
Research algorithm

The present study seeks to optimize the inventory and distribution in the blood logistics chain. The first part of the model, which is implemented in the RBC in the region, uses a combination of simulation algorithm and neural network. This data includes amount of the frequency of donation (blood collection rate) of each CBC, the amount of inventory at the beginning and end of the period, the amount of waste (separated by donation and laboratory waste), the amount of return due to non-use and expiration in the period, the amount of blood that consumers returned to suppliers due to the expiration and non-use, and the amount of blood that each supplier sent to each consumer individually during the time period. For red blood cells, it is daily according to blood groups A, B, O, and AB. The output of this algorithm determines the type and behavior of the inventory system at the level of CBC and hospitals, which is communicated to them annually by RBC. After communicating the type of inventory management system to the lower levels, they are required to implement this policy. The CBC level, by implementing the announced policy, should take over the distribution of blood to hospitals in order to complete the optimization of the conditions of this chain. To this end, the multi-objective planning technique has been used. Figure 3 shows the research algorithm.

To implement the developed model, the following procedures are followed in detail:

1. The initial level of inventory management optimization in the region should involve changing consumer ordering patterns and supplier inventory behavior. RBC assign coordination and supervision of CBC performance. As a result, an RBC policy needed to be prepared and communicated. In order to do this, a model that implements the S and R inventory control policy and necessitates the entire region to implement it was developed with the use of a simulation algorithm and neural network. S represents for the target inventory amount, while R refers to reorder point. In order to reduce waste and shortages in each database (including each CBC and each hospital), the simulation algorithm is used in this stage to determine the optimal amount of the target inventory and the reorder point. The determined values are then conveyed to the CBCs so they may develop and implement the appropriate instructions to work with their consumers (Fig. 3, RBC level).
2. They develop the necessary plan to provide their consumers with the optimal number of shipments each day. The manager of each CBC’s blood bank need to select the appropriate amount of blood to be sent to their consumers in accordance with the routing plan, taking into account the amount of stock in his central base and the overall amount of demand from the hospitals included by his coverage (Fig. 3, CBC level).
3. After receiving blood from the transporter, hospitals, as consumers, add blood to their inventory. After giving it to the patients, the colleagues compare the remaining stock to the reorder point at the end of the day and decide to place an order the following day (Fig. 3, hospitals level).

The optimal distribution and inventory management methods at the three levels of RBC, CBC, and hospitals are described in the sections that follow.

#### Designing inventory management model by RBC organization

To reduce the amounts of deficiencies and wastes in the system under investigation, RBC designed the inventory management model with the aim of identifying the target inventory values and the ordering point of each supplier and consumer in the network. As a result, this model was developed using a simulation technique at the RBC level, the output of which will be instructions for the CBC that specify the target inventory values and the ordering point in each supplier. The parameters linked to the implementation of the simulation model were stored in a database using the MATLAB program, and the output results were recorded. It is essential to accurately estimate consumer demand, which is accomplished with a neural network, as the input of the simulation model requires information on demand. As a result, the explanation of the simulation flowchart follows the explanation of anticipating consumer demand.

The following assumptions and limitations have been considered while implementing the model:

- Wastes are divided into two categories: wastes resulting from blood expiration and wastes from non-use of blood. Additionally, each frequency is individually entered into the model
- The supply of blood is subject to fluctuating demand.
- For each hospital and CBC, a specified initial blood stock level is established.
- Specific precautionary stock levels are set for every CBC and hospital.
- Blood may be transferred between CBC.
- The region of the model is considered to consist of 54 hospitals and six CBC.
- A shortage penalty for the supplier is taken into consideration due to the high level of uncertainty in the demand.
- A regional network utilizes a centralized structure
- The circular policy is the one employed by the regional network. The blood is transported to the hospital as part of the circular system, but the blood bank at the CBC is still in charge of the blood units can transport them to other hospitals. In other words, goods can be sent to the main blood transfusion center.

##### Estimating consumer demand using neural network

The CBC often send less blood than requested because they are aware of the fact that daily consumers in the network under study declare their needs to suppliers in addition to what they want. The daily demands of each consumer must be taken into account while implementing the inventory model using the simulation technique. As a result, in order to provide the other steps of the inventory management model and the required inventory values, it was essential to first determine the value of each consumer’s demand. In other words, the method contrasts what is really sent with what is recorded in the present inventory management system as consumer demand.

**Fig. 4:**
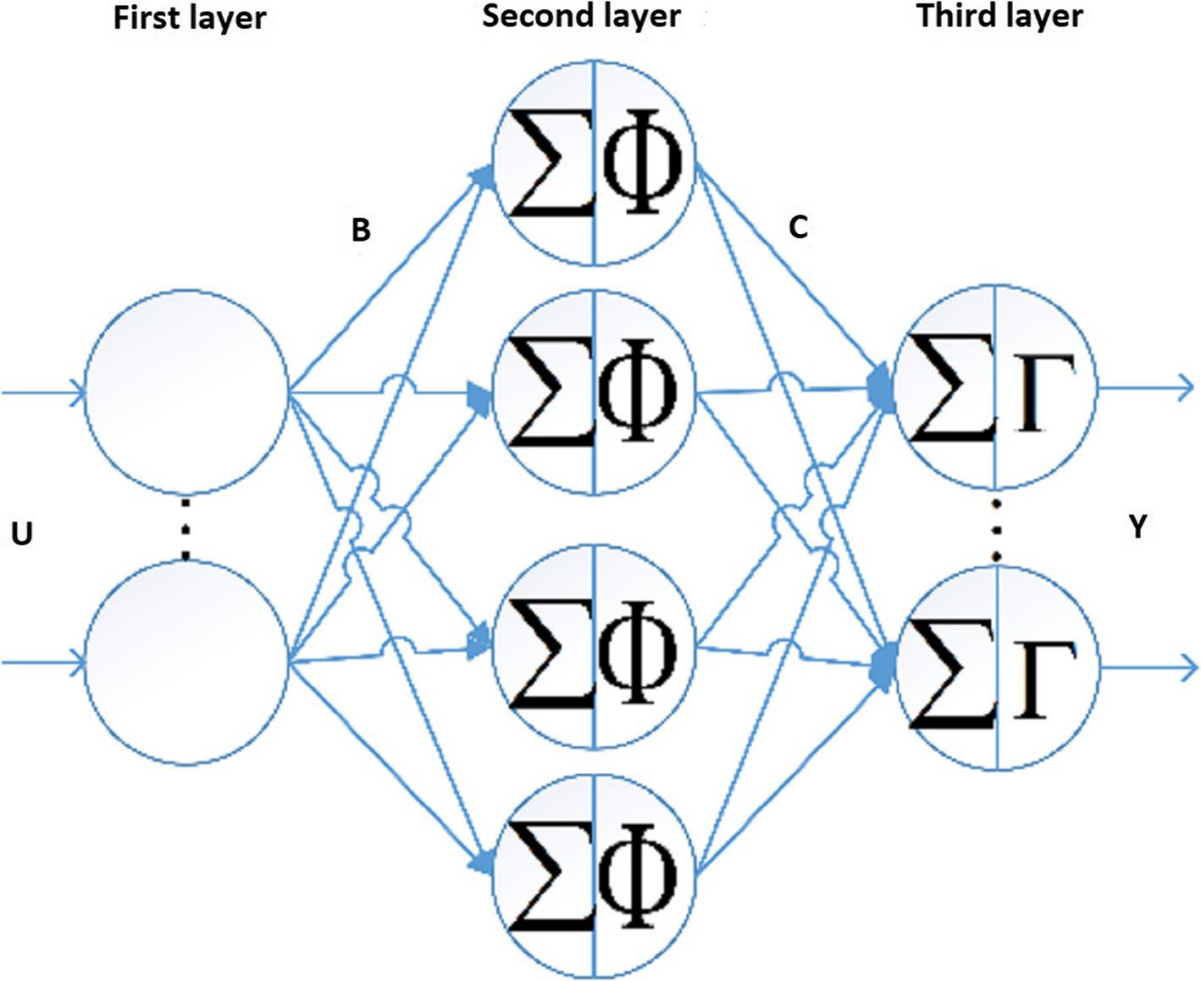
Feedforward neural network

Here, Feedforward Neural Network is used to find the non-linear mapping in question. The Feedforward Neural Network consists of three layers (Fig. 4). The first layer is the input layer; the input enters the hidden layer after passing through the first layer; this layer consists of one to several sub-layers. Then the output of the hidden layer enters the output layer and finally the output appears. $U, Y, B, C\in {\mathbb{R}}$ are input data, output data, weight between input and hidden layer, and weight between hidden layer and network output, respectively. Γ is the activation function of the output layer and Φ is the activation function of the hidden layer. Figure 4 shows the Feedforward Neural Network.

The relations governing the feefforward neural network are as follows:

1 $$Z=\varnothing \left[UB\right]$$

2 $$Y=\Gamma \left[ZC\right]$$

3 $$E=Y-{Y}^{e}$$

4 $$\Delta B=\frac{\partial Y}{\partial B}= {\Gamma }^\prime\left[Y\right]C{\phi }^\prime\left[U\right]E$$

5 $$B\left(k+1\right)=\Delta B+B\left(k\right)$$

6 $$\Delta C=\frac{\partial Y}{\partial C}={\Gamma }^\prime\left[Y\right]ZE$$

7 $$C\left(k+1\right)=C\left(k\right)+\Delta C$$

where E is the error of the actual value with the value estimated by the neural network. ΔC and ΔB are the changes weights during the training process.

**Fig. 5:**
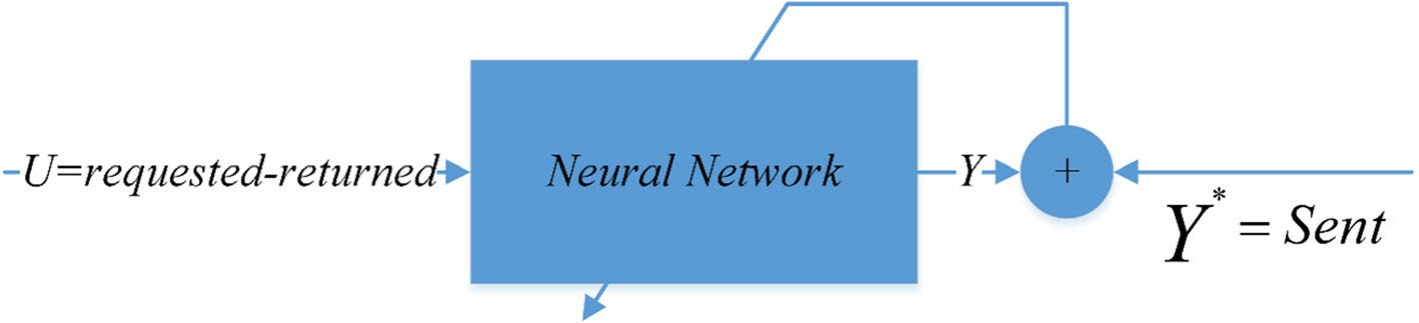
Relationship between sent and requested blood units

The purpose of this study is to determine the link between the amount of blood units delivered in response to hospital and other blood transfusion requests and the amount of blood units that are returned. U is the sum of requests less returns, where Y is the number of submissions. Figure 5 illustrates this relationship. This method prevents the blood units from expiring as a result of non-use by including the number of returns as a penalty amount in the calculations.

The data is initially gathered and divided into two sets of input and output data sets. This was followed by the establishment of the number of neurons in each layer, network parameters, net input, transfer function, learning function, learning rate, and learning function. After that, the data was normalized and standardized for preprocessing. For the next stage, the data was divided into three categories training, test, and validation data. Finally, the neural network was used, and the necessary modifications were made to achieve the best possible outcomes. Each consumer’s demand from Eq. 8 was taken into account as the input layer variable for the neural network. The blood request on day t is decreased by the total returns for non-use and returns for each hospital’s expiration on day t.

This equation requires the value of returns to be included in the calculations as the penalty amount, preventing the expiration of blood units due to non-use. The output layer predicts the daily requirement for red blood cells. Based on mean squared error, the right number of neurons in the hidden layer has been chosen (MSE). The input layer, the hidden layer, and the output layer are fully interconnected. The connection weights are chosen at random by the algorithm, and they are modified as it gains knowledge. By taking into consideration the sigmoid in the hidden layer, Eq. 9 is utilized to calculate the sigmoid of the weights in the range [-1,1].

8 $${A}_{tir}={D}_{tir}-{C}_{tir}-{O}_{tir}$$

${D}_{tir}$: Hospital i blood request for blood group r day t

${C}_{tir}$: The returns due to non-consumption of hospital i for blood group r day t

${O}_{tir}$: The rate of return due to hospital expiration i for blood group r day t

${A}_{tir}$: Consumer i demand for blood group r day t

9 $$f\left(t\right)=\frac{2}{1-{e}^{-2t}}-1$$

The simulation algorithm is then applied to the distribution of the expected hospital requests, the value of each central base’s consumption (demand), the range of target inventory values, the hospital and central base reorder points, and the lifespan of the product. The average weekly (daily) demand for each blood type has been calculated separately by each central database and hospital, and 20% of this value is utilized to define the range of target inventory values for each database. Five of the calculated average daily re-ordering points for each central and hospital base per blood type were used to determine the maximum and minimum interval values. Additionally, each database’s inventory data was gathered everyday while ordering throughout the course of a year in order to identify the range of re-ordering points. It’s important to keep in mind that the simulation algorithm has already been given the appropriate intervals. The interval is reduced if the proposed algorithm’s optimal value during implementation is on the minimum interval value, while the period is considered longer if it is on the maximum interval value.

The complete amount of data for each type of red blood cell from September 2015 to September 2017 was then divided into 230 days for network testing and 500 days for training and verifying the neural network. A variety of network architectures with the number of hidden layers (between 2 and 15 neurons), three input variables, and one output variable were looked at in order to select the appropriate neural network model to predict the blood demand of each user by blood group (Table 2). The model with the lowest MSE value was ultimately chosen.

**Table 2:** The best neural network model for various types of red blood cells according to MSE criteria

**Specifications**	**A**	**O**	**B**	**AB**
**Network architecture**	**(3:5:1)**	**(3:7:1)**	**(3:9:1)**	**(3:8:1)**
**MSE**	**0.013**	**0.0092**	**0.0099**	**0.0072**

The MSE function calculates the mean square of the differences between the actual and predicted values. Squaring the magnitude of the differences causes larger errors to receive more error than smaller errors; this means that the model is penalized for its big mistakes. By selecting a lower MSE, you may avoid the expiry of blood units as a result of non-use because the amount of returns is treated as a penalty in the proposed model.

##### Determining the optimal ordering policy of CBC level and hospitals using simulation

**Fig. 6:**
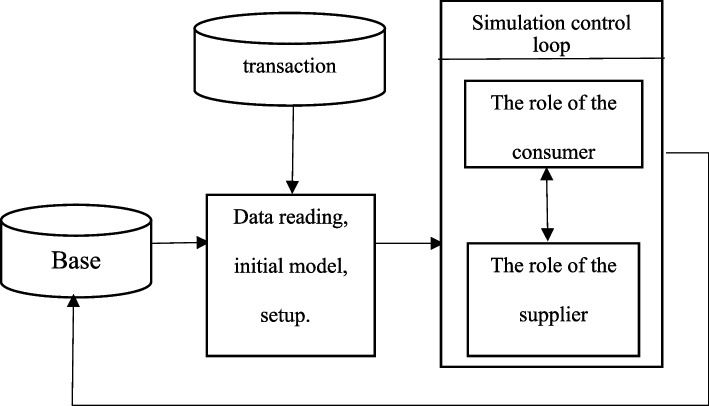
Schematic representation of the structure of the simulation framework

In order to determine the best inventory management system using the simulation technique, RBC needs the demand distribution function of each database (central bases and hospitals), as was previously discussed. For the reasons described in the preceding section, the neural network method was also employed to determine each consumer’s actual demand. Next, the range of target inventory values, the reorder point of each central base and hospital, the lifespan of the products, and the distribution function of the estimated demands of the hospitals and the amount of consumption (the amount of demand) of each central base are entered into the reusable simulation algorithm. A database for archiving past data, a data transaction level that shows process details from the point of data collection through preparation at the supplier’s location, and an object-oriented simulation model developed in Microsoft make up the reusable simulation framework in this study. The schematic representation of the framework structure for this type of simulation is shown in Fig. 6.

The characteristics of each central base and hospital’s demand distribution function are described in the sections that follow, along with directions on how to calculate the range of target stock values and the reorder point for each of them. It is important to note that the symbol i has been allocated to each central base and hospital in the network. Six CBC and 54 hospitals were included in the case study, hence 60 databases were required to use the simulation technique. Additionally, each database has been implemented independently for each of the four blood groups as the study is focused on these four major blood types. As a result of the large number of them, only the distribution function, target inventory intervals, and central bases’ reordering point are discussed in this section. Distribution function of central base in shown in Table 3.

**Table 3:** Distribution Function of CBC

**CBC**	**i**	**Distribution Ffunction**			
		**A**	**O**	**B**	**AB**
Mashhad	1	N^a^	N^a^	N^a^	N^a^
Sabzevar	2	P^b^	P^b^	P^b^	P^b^
Torbat Heydarieh	3	P^b^	P^b^	P^b^	P^b^
Gonabad	4	P^b^	P^b^	P^b^	P^b^
Neyshabur	5	P^b^	P^b^	P^b^	P^b^
Ghuchan	6	P^b^	P^b^	P^b^	P^b^

^a^Normal distribution function

^b^Poisson distribution function

The average weekly (daily) demand of each central base and hospital was determined by blood group in order to set the range of target inventory values for each CBC, and 20% of this value was used as the maximum and minimum values of the range. Additionally, each database’s stock information was gathered every day for a year in order to establish the reorder point interval. Additionally, the average daily reorder point for each CBC and hospital has been calculated by blood group, and the maximum and minimum values of the interval have been set at 5 of this value. It should be noted that the desired intervals are incorporated in the simulation algorithm; thus, the interval will change depending on whether the optimal value suggested by the algorithm is placed on the minimum or maximum value of the interval during implementation. Table 4 provides an illustration of the range values of the target inventory amount and the reorder point of the CBC.

**Table 4:** The values of the target inventory range and the reorder point of the CBC

**Central Base**	**i**	**Target inventory values**			
		**A**	**O**	**B**	**AB**
Mashhad central	1	$160\le S\le 240$	$152\le S\le 228$	$120\le S\le 180$	$52\le S\le 78$
Sabzevar central	2	$64\le S\le 96$	$56\le S\le 84$	$24\le S\le 36$	$26\le S\le 39$
Torbat Heydarieh central	3	$64\le S\le 96$	$56\le S\le 84$	$32\le S\le 48$	$20\le S\le 29$
Gonabad central	4	$24\le S\le 36$	$24\le S\le 36$	$20\le S\le 30$	$16\le S\le 24$
Neyshabur central	5	$24\le S\le 36$	$16\le S\le 24$	$16\le S\le 24$	$15\le S\le 22$
Ghuchan central	6	$24\le S\le 36$	$16\le S\le 24$	$16\le S\le 24$	$12\le S\le 18$
**Central base**	**i**	**Reorder point values**			
		**A**	**O**	**B**	**AB**
Mashhad central	1	$15\le R\le 25$	$13\le R\le 23$	$5\le R\le 15$	$3\le R\le 13$
Sabzevar central	2	$5\le R\le 15$	$5\le R\le 15$	$0\le R\le 10$	$1\le R\le 9$
Torbat Heydarieh central	3	$5\le R\le 15$	$5\le R\le 15$	$0\le R\le 10$	$0\le R\le 10$
Gonabad central	4	$0\le R\le 10$	$0\le R\le 10$	$0\le R\le 7$	$0\le R\le 7$
Neyshabur central	5	$0\le R\le 10$	$0\le R\le 10$	$0\le R\le 7$	$0\le R\le 7$
Ghuchan central	6	$0\le R\le 10$	$0\le R\le 10$	$0\le R\le 7$	$0\le R\le 7$

To perform the simulation technique, it is necessary to determine the number of iterations and the length of repetitions of each performance in each iteration. Various demands are produced and measured from Eq. 10 (formula t-test), and the number of repetitions of each simulation run was determined according to the distribution of demand or consumption of hospitals and central bases. The error percentage numbers (B) and standard deviation (s) are calculated using this equation, and the value of the variable n (number of repetitions of each execution) is calculated using the t-test procedure. The average value of the objective function is (TC) (minimizing the number of wastes and shortages).

10 $$\left[\overline{TC }-{t}_{n-\mathrm{1,1}-\frac{B}{2}}\frac{s}{\sqrt{n}},\overline{TC }+{t}_{n-\mathrm{1,1}-\frac{B}{2}}\frac{s}{\sqrt{n}}\right]$$

The number of iterations for each execution implementation is 20, taking into account the standard deviation and error rate of 5%. Twenty iterations were therefore performed for each simulation run over the course of a year (iterations length). According to Fig. 5, the implementation algorithm for each simulation run is carried out independently for each central base, hospital, and blood group. This figure’s top part is in charge of managing inventory and meeting demand. The consumer enters the system with a specific distribution function, in accordance with the method depicted in Fig. 5. If there is an inventory or not, one must check it when the consumer enters If _IL_ri_ > 0, the amount of missing values is increased by one unit. However, the blood units are provided to the customer if IIL_ri_ > 0 (deplet). Deplet + + indicates that the number of consumers whose demand has been fulfilled is calculated at this point. One must determine if the instantaneous inventory level (ILri) has reached the R_ri_ level or not when blood units are removed from the system. Nothing is done if the Rri level is not achieved (dispose). But in order for the inventory level to rise to the s_ri_ level if it has already reached the R_ri_ level, an order must be placed. In this instance, the number of blood orders is increased. When the product life is finished, the lower part of the algorithm is in charge of removing expired blood from the system. The algorithm decides that once the inventory level reaches this value, the inventory managers access the system at least once every day to clean up corrupt materials, check how much blood has been consumed by consumers, and how much of the target inventory has expired. It is calculated by allowing lifespan of the product (after several days, the inventory manager checks in and removes products that have expired).

**Fig. 7:**
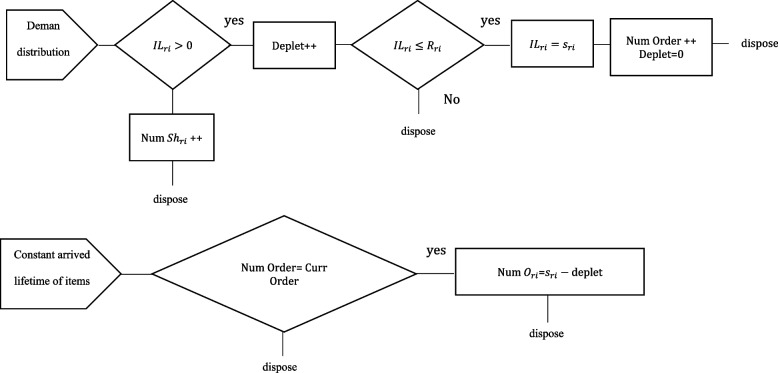
Simulation algorithm

As a result, the algorithm in Fig. 7 defines a continuous inventory policy for the system’s hospitals and central bases. When the inventory level of each role reaches the reordering point ri, the inventory system of that role (Supplier or Consumer) raises its inventory level to the target inventory level Si - orders the value of Si-ri (such as ${r}_{i}\le {S}_{i}$). This process is repeated for each of the central bases and hospitals after the target inventory values and reordering points have been established. In order to reduce the amount of waste and shortage throughout the whole system, the simulation algorithm analyses all supplied intervals and provide the optimal target inventory and reorder point for each consumer and supplier role. The last element of the method serves as a repository for the information produced by the simulation as well as a place to store the data needed to operate the model. The symbols used in the simulation technique are listed in Table 5.

**Table 5:** The symbols of simulation technique

**Symbol**	**Definition**
${UI}_{ir}$	Consumption distribution (demand) function of central base i for blood group r
${UA}_{ri}$	Hospital demand distribution function i for blood group r
${s}_{ri}$	Target inventory level of each consumer and supplier i in blood group r
${R}_{ri}$	Reorder point of each consumer and supplier i in blood group r
${LF}_{r}$	Shelf life of products
${s}_{ri}^{*}$	The best level of inventory of the target hospital or central base i in blood group r
${R}_{ri}^{*}$	Reorder point of each consumer and supplier i in blood group r
${Sh}_{ri}$	Number of deficiencies of each consumer and supplier i in blood group r
${O}_{ri}$	Number of expired blood consuming or supplying i for blood group r

#### Implementation of inventory management model on a daily in CBC’s and hospitals

The CBC are required to implement the inventory management model in accordance with the provided instructions after being informed of the target inventory values and reorder point. Daily activities are conducted from the supplier to the consumer to implement the inventory management model. The blood bank resource manager of the supplier determines the blood bank reservoir balance at the outset of the day. This quantity of inventory contains all of the healthy blood that has been collected from donation bases (both mobile and fixed) and transported to the CBC for the planned tests, as well as any remaining blood from earlier days. Blood received from consumers with extra inventory and sent to the returned supplier tank is deducted from the value of blood in the tank which has expired. Additional blood received from other CBC, residual blood from previous days with a shelf life, and surplus blood received at the outset of the day from other CBC are also considered. The value of each consumer’s demand is obtained independently in the next step (the demand is received from RBC for the first time, but for the next time, the order is directly taken from the consumer). The CBC Inventory Manager determines if the total demand is less than the target inventory stated by the RBC after calculating the total demand received. All requests will be fulfilled if they have already been all completed and have not yet reached the desired balance. However, the inventory management must determine the value provided to each consumer if the value of the requests exceeds the value of the target inventory. It will send consumers’ blood and divide it among them in accordance with the routing model to speed up delivery and reduce shipping costs. This is carried out in accordance with Designing inventory management model by RBC organization section’s description of multi-objective planning. If it receives emergency orders after sending requests to consumers in accordance with the routing model, it will respond to them up to the end of the day, and at that point, it will once again calculate the blood bank’s balance. It sends the surplus to other central inventories in the network if the remaining balance is higher than the target inventory and demands blood from other CBC if the remaining balance has reached the reorder point. The supplier day (CBC) ends here. The following is planned for simultaneously with the provider of consumer behavior (hospitals) throughout the day. It receives the required blood from the supplier, adds it to its inventory, and progressively uses it during the course of the day. The hospital blood bank manager computes his balance at the end of the day. In order to keep the blood bank’s balance from increasing and developing into a perishable good, it is sent to its supplier if it surpasses the target balance set by the CBC (Commodity return). If the current inventory is less than the stated target inventory, it is checked to see if this inventory has reached the reorder point or not. It will decide the desired order amount for the following day if it reaches the re-order point; otherwise, everything occurs, and the day comes to an end.

The CBC are required to implement the inventory management model in accordance with the provided instructions after being informed of the target inventory values and reorder point. Daily activities are conducted from the supplier to the consumer to implement the inventory management model. The blood bank resource manager of the supplier determines the blood bank reservoir balance at the outset of the day. This quantity of inventory contains all of the healthy blood that has been collected from donation bases (both mobile and fixed) and transported to the CBC for the planned tests, as well as any remaining blood from earlier days. Blood received from consumers with extra inventory and sent to the returned supplier tank is deducted from the value of blood in the tank which has expired. Additional blood received from other CBC, residual blood from previous days with a shelf life, and surplus blood received at the outset of the day from other CBC are also considered. The value of each consumer’s demand is obtained independently in the next step (the demand is received from RBC for the first time, but for the next time, the order is directly taken from the consumer). The CBC Inventory Manager determines if the total demand is less than the target inventory stated by the RBC after calculating the total demand received. All requests will be fulfilled if they have already been all completed and have not yet reached the desired balance. However, the inventory management must determine the value provided to each consumer if the value of the requests exceeds the value of the target inventory. It will send consumers’ blood and divide it among them in accordance with the routing model to speed up delivery and reduce shipping costs. This is carried out in accordance with Designing inventory management model by RBC organization section’s description of multi-objective planning. If it receives emergency orders after sending requests to consumers in accordance with the routing model, it will respond to them up to the end of the day, and at that point, it will once again calculate the blood bank’s balance. It sends the surplus to other central inventories in the network if the remaining balance is higher than the target inventory and demands blood from other CBC if the remaining balance has reached the reorder point. The supplier day (CBC) ends here. The following is planned for simultaneously with the provider of consumer behavior (hospitals) throughout the day. It receives the required blood from the supplier, adds it to its inventory, and progressively uses it during the course of the day. The hospital blood bank manager computes his balance at the end of the day. In order to keep the blood bank’s balance from increasing and developing into a perishable good, it is sent to its supplier if it surpasses the target balance set by the CBC (Commodity return). If the current inventory is less than the stated target inventory, it is checked to see if this inventory has reached the reorder point or not. It will decide the desired order amount for the following day if it reaches the re-order point; otherwise, everything occurs, and the day comes to an end.

##### Designing distribution management model

To improve the blood logistics chain’s transport status, a routing model was developed. The above model will implemented, giving each CBC better control over how blood is distributed while also preventing potential waste in the transport machine and the clogging of applicant machines at once. Choosing the best route model with the lowest cost and loss is the objective of this stage.

The multi-objective programming mathematical model that served as the study’s routing model is represented by its symbols in Table 6. The model’s basic assumptions and specifications are as follows:

- There is blood exchange between central bases (CBCs) in the logistics network.
- In a logistics network, a supplier delivers blood to multiple consumers (hospitals under its cover and other central bases).
- It is impossible to exchange blood units between hospitals.
- The supplier uses a heterogeneous transport network (fleet) with vehicles that differ in capacity and cost to distribute blood to consumers.
- Each blood unit has a specific expiration date and after that it will be unusable.
- It is possible for consumers to return expired or unused blood.
- Consumer requests have to be submitted within a specified time frame.
- The inventory capacity of each consumer for each blood is recognized by the supplier.
- A cost of shortage penalty is considered for the supplier because of the high degree of uncertainty in the demand value.
- The level of initial blood supply in each consumer is known.
- Precautionary reserve is specified for each supplier and consumer.

**Table 6:** Routing technique symbols

**Symbol**	**Definition**
${c}_{ij}$	The distance between the consumer i and j
${\widetilde{\upsilon }}_{ijk}$	The cost of transferring each unit between consumer i and j for vehicle type k
${cap}_{k}$	The capacity of vehicle type k
${A}_{tri}$	Consumer demand i from blood group r on day t
M	Big number
${M}{\prime}$	Database and consumers collection
${{\varvec{x}}}_{{\varvec{i}}{\varvec{j}}{\varvec{k}}{\varvec{t}}}$	It gets 1 if consumer j is served by device k in period t exactly after consumer i;
${z}_{ikt}$	It gets 1 if consumer i is served by device k in period t, otherwise 0
${q}_{irkt}$	The amount of blood group r received by consumer i by device k over time t
${w}_{ikt}$	The artificial variable shows the nth consumer served by the truck.

The primary objective function of the model is to reduce transportation costs. Equation 4 represents this function’s mathematical form. Equation 5’s second objective function is to reduce total distance traveled. The sixth constraint ensures that each vehicle’s capacity should not be overly particular. This limitation indicates that the supplier must be the vehicle’s initial starting place (CBC). Vehicle turning is represented by Eq. 7. Equation 8 is used to exclude the sub-tour from the route graph so that a car will not return to a consumer. This constraint demonstrates that ${w}_{jkt}$ must be greater than ${w}_{ikt}$ in order to prevent the vehicle from serving the same consumer more than once and from modifying the graph path by going from consumer I to consumer j and serving them (there is a connection between consumers I and j in direction I to j). The decision variables are shown in Eqs. 11 and 17.

11 $$Min {f}_{1}= \sum\nolimits_{(i,j)\in {M}^\prime}\sum\nolimits_{k\in K}\sum\nolimits_{t\in T}{\widetilde{\upsilon }}_{ijk}{y}_{kt}{x}_{ijkt}$$

12 $$Min {f}_{2}= \sum\nolimits_{(i,j)\in M}\sum\nolimits_{k\in K}\sum\nolimits_{t\in T}{{c}_{ij}x}_{ijkt}$$

13 $$\begin{array}{cc}\sum_{r\in R}\sum_{i\in M}\sum_{t \in T}{q}_{irkt}\le {cap}_{k}{y}_{kt}{z}_{0kt}& \forall k, t\end{array}$$

14 $$\begin{array}{cc}\sum_{j\in {M}^\prime}{x}_{ijkt}=\sum_{j\in {M}^\prime}{x}_{jikt}={z}_{ikt}& \forall i\in {M}^\prime, k, t\end{array}$$

15 $$\begin{array}{cc}{w}_{ikt}-{w}_{jkt}+\left(\left|M\right|+1\right){x}_{ijkt }\le M& \forall (i,j)\in M, k, t\end{array}$$

16 $$\begin{array}{cc}{x}_{ijkt},{z}_{ikt}\in \left\{\mathrm{0,1}\right\}& \forall i,j\in M, k, t, i\ne j\end{array}$$

17 $$\begin{array}{cc}{q}_{ikt},{I}_{irt},{EX}_{irt},{SH}_{irt},{y}_{kt}\ge 0,& Integer \forall i\in M, r, k, t\end{array}$$

The routing model seeks to identify the optimal path for blood delivery to consumers with the potential of returning blood to suppliers as part of a multi-objective planning model for reducing transportation costs and reducing the distance traveled. Cplex software was used to solve the model.

The epsilon constraint method was applied to reduce the multi-objective function to a single-objective function. This approach is one of the widely used methods for solving multi-objective problems. It does so by shifting all of the objective functions at each step, except for one, to the constraint. The constraint method may be used to define the boundaries of your fragment.

## Results

The Khorasan Razavi blood transfusion network used the study’s suggested model, which had a structure similar to Fig. 4, and ran it. The blood transfusion network of Khorasan Razavi implemented the proposed research model in a comprehensive and integrated manner. Since the model used in this study has two primary elements management and routing—each of the results was obtained separately from the province’s central database and the structures of the model’s two main components. Hoewever, in this instance, we will only express the central database’s research results. It is said that Mashhad is the main base. Six hospitals in the province’s cities without a central base and 27 hospitals in Mashhad are continually served by the base.

### The results of implementing optimal inventory management model for Mashhad central base

The 27 hospitals in Mashhad and the six hospitals in the cities without a central base are continually served by this base, which is the city of Mashhad’s central blood transfusion base (Sarakhs, Fariman, Chenaran, Dargaz, Taibad, Torbet-e Jam). There is an exchange of sending and receiving blood amongst the central bases of the province, with Sabzevar, Torbat-e Heydarieh, Gonabad, Neishabur, and Quchan serving as both a supplier and a consumer for the other central bases. In Razavi Khorasan Province, this site is regarded as the largest central blood transfusion base. Consumers of this database are divided into two categories in order to simplify the display of results: Mashhad consumers (hospitals situated in Mashhad) and city consumers (hospitals and central centers of the provinces).

The optimal amount of the target inventory and the reorder point in the central base of Mashhad and hospitals it covers have all been discussed as results of the implementation of the inventory management model. Hospital demand estimation variables and their maximum order amounts have also been discussed.

#### Estimation of the demand of hospitals

In order to estimate the real demand of the hospitals in this base, the data was analyzed in three ways.

**Fig. 8:**
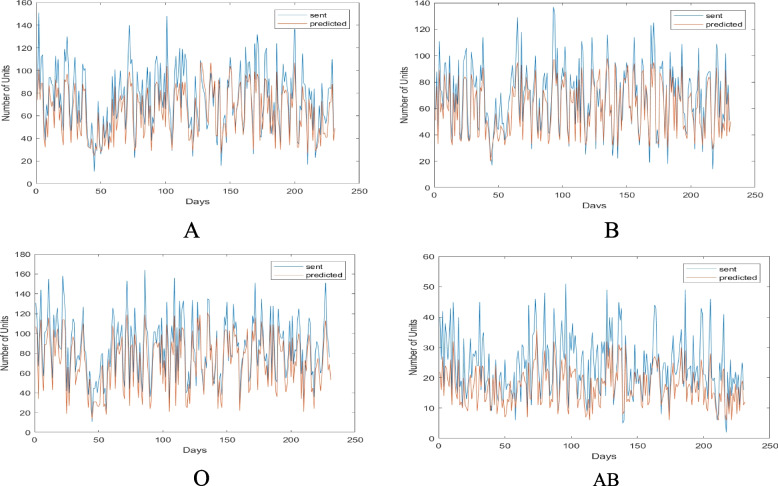
The values of actual demand and blood transfusion to Mashhad hospitals of Mashhad central base by blood group

In the first method, the total demand of consumers (Mashhad hospitals and other cities covered by it) was divided based on the types of blood group and the type of consumer, and their demand was evaluated using the neural network technique in the 230-day test period. The values of the actual demand and delivery of blood to Mashhad database consumers are shown in Fig. 8 by blood group. The red lines in Fig. 8 represent the optimal and actual consumer demand from Mashhad’s central base. The results in this graph demonstrate that the Mashhad hospitals would need to request the required blood units in accordance with the red lines if they were to order the optimal amount to prevent shortages and waste. The value of blood requested by each hospital in Mashhad from the city’s central base is shown by the blue lines for each blood group. The gap between the current and desired states is indicated by the difference between the blue and red lines. In fact, as seen by the various blood types, Mashhad’s hospitals sought significantly more blood than they actually needed, which resulted in enormous system waste in the form of returned blood. On certain days, the blue lines were far more than the red lines, indicating significant blood loss across the logistics chain. In the uncommon blood type O, this much error and the difference between what was meant to be requested and what really occurred can cause irreparable damage. There is a critical requirement for effective inventory planning and strict consumer monitoring since the database is more sensitive than other databases in the area due to the vast number of consumers.

**Fig. 9:**
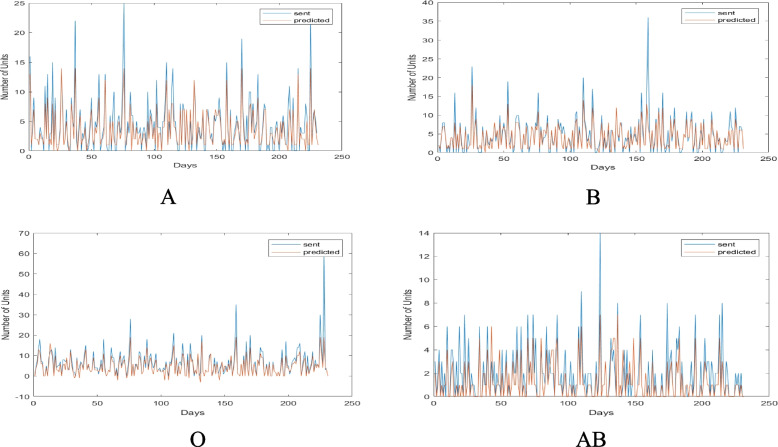
Amounts of actual demand and delivery of blood to the hospitals of Mashhad central base by blood group

In the second method, the total demand of the consumers was divided into different blood groups and was calculated separately for each consumer over the course of 230 test days. Figure 9 displays the actual blood delivery and demand in this city, subdivided by blood group. The trend of the red and blue lines in this graph indicates that the city hospitals serving this base of blood group O patients have operated almost effectively, and except for a small number of cases when they ordered less blood than was necessary, they have generally been close to optimal. However, in the case of the AB blood group, they frequently ordered less than what was required, which caused a shortage and put patients at danger for irreversible damage. According to the findings in this graph, the hospitals in the cities should have requested the blood units they need in accordance with the red lines if they wished to place their orders in the most optimal way to prevent shortages and waste. The base is more sensitive than other bases in the area due to the high number of consumers; as a result, it has a critical need for careful consumer monitoring and optimal inventory planning.

**Fig. 10:**
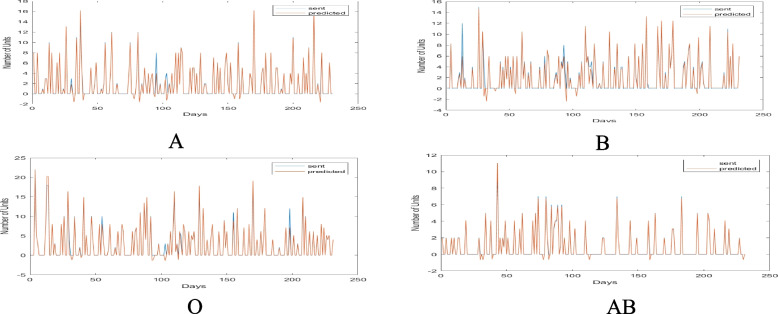
Amounts of actual demand and blood supply sent to Aria Hospital divided by blood type

Figure 10 displays the actual demand amounts given to consumers of this base by blood type and consumer for Aria Hospital as an example of a Mashhad consumer.

The results of this figure allow us to draw the conclusion that, in actuality, Aria Hospital received requests on some days that were far more than what they needed, and on other days that were significantly less. This will cause significant waste and shortages throughout the whole system. What can be inferred from the results of this hospital is that it frequently requested blood in blood types A and O in surplus of what was actually needed. As a result, there will be a lot many emergency orders, which will cause shortages at the hospital. As a result, by implementing the proposed model, it will be feasible to significantly reduce waste and shortages while also maintaining enough supervision of hospitals’ performance in terms of the number of orders and their consumption.

#### Maximum order quantity of each consumer

It is feasible to estimate the maximum amount of each consumer’s order based on the prediction of their demand from each base. The findings of this estimate for Mashhad’s consumers and the city of its central base are calculated in accordance with Table 7.

**Table 7:** The maximum order amount of Mashhad central base hospitals by blood group

**Aria**				**Naja**			
**A**	**O**	**B**	**AB**	**A**	**O**	**B**	**AB**
5.9033	6.2099	6.3102	9.5644	13.375	12.3732	11.894	4.96506
**Artesh**				Javad Alaima Tabarsi			
**A**	**O**	**B**	**AB**	**A**	**O**	**B**	**AB**
5.6583	5.3546	5.7140	6.7367	7.0481	7.0481	4.4305	4.1943
**Imam Zaman**				**Dr. Sheikh**			
**A**	**O**	**B**	**AB**	**A**	**O**	**B**	**AB**
10.9070	11.1917	11.7953	6.3161	26.209	14.8823	11.63375	8.8947

#### The results of the target inventory of Mashhad central base and the hospitals covered by it

According to the demand distribution function of Mashhad central base and its consumers, as well as the shelf life of the sent bloods, the simulation technique has determined their target inventory with the minimum waste and shortage with the results of Mashhad central base and several hospitals given as an example in Table 8. In other words, each central base and hospital has the maximum inventory it can hold that ends in the least waste and shortage.

**Table 8:** Target inventory amount of Mashhad central base and some hospitals covered by it (for instance)

**Database**	**Target Inventory Amount**			
	**AB**	**B**	**O**	**A**
Mashhad Central Base	65	132	200	195
Aria Hospital	3	6	8	7
Artesh Hospital	2	3	4	3
Imam Zaman Hospital	3	7	12	9
Javad Al-aemeh Tabrasi Hospital	1	2	3	3
Dr.Sheikh Hospital	4	12	16	10

#### Reorder point

At the beginning of the developed model, by using a combination of neural network and simulation, the demand of each blood group is predicted by each user of the database. At the end of the model, if the demand is made according to the calculated forecast, the reorder point of each base and the hospitals covered by it is obtained by considering the minimum waste and return. Table 9 shows the reorder point values of this database and its consumers.

**Table 9:** The reordering point of Mashhad central base and some hospitals covered by it (for instance)

**Reorder Point Value**				**Database Name**
**A**	**O**	**B**	**AB**	
20	15	8	5	Mashhad CBC
2	2	2	1	Aria Hospital
1	1	1	1	Artesh Hospital
2	3	3	1	Imam Zaman Hospital
1	1	1	1	Naja
4	4	4	1	Dr. Sheikh Hopsital

### The results of implementing the routing model of Mashhad central base

The actual demand calculated by the neural network, the value of the target inventory, and the reordering point of the distribution and routing simulation output for seven days of Mashhad Central Base were compiled in accordance with the inventory data at the start of the period. In doing so, seven days were taken into account between October 2015 and October 2017, and a routing plan was developed based on the Mashhad Central Base inventory at the beginning of the period, the estimated real demand of this base’s consumers during those seven days (as determined by neural network output), the value of the target inventory, and the point of re-ordering it and its consumers. The Cplex software is used to solve this model, and a Pentium Corei5 processor with a clock frequency of 2.27 GHz and 4.0 GB of RAM is required. As a result, Table 10 is used to illustrate the vehicle information that was obtained from the distribution base of the main base.

**Table 10:** Information of the vehicle used in the routing model

**Vehicle types (K)**	**Vehicle number**	**Capacity of vehicle type k (cap**_**k**_**)**
Small	3	90
Medium	2	150
Large	1	300

The planning period in this model is seven days, and Table 11 shows the distribution route for the first day of the planning period for the consumers of this base (consumers from Mashhad and the cities).

**Table 11:** Blood distribution route among the users of Mashhad central base on the first day

**Consumers of Mashhad**				
**Vehicle type**	**Distribution route**	**Consumers**	**Blood type**	**The blood received (Q)**
1	Central base, Ghaem, Imam Reza, Central base	Ghaem	A	3
			B	0
			AB	2
			O	7
		Imam Reza	A	10
			B	3
			AB	6
			O	5
2	Central base, Javad Al-aeme Tabrasi, Sina, Central base	Javad Al-aeme Tabrasi	A	5
			B	1
			AB	0
			O	7
		Sina	A	0
			B	0
			AB	0
			O	5
3	Central base, Taleghani, Shahid Hasheminejad, Central base	Taleghani	A	0
			B	0
			AB	0
			O	2
		Shahid Hasheminejad	A	2
			B	8
			AB	4
			O	4

## Validation

The validation is done independently for each step as described below since the model is divided into three sub-stages.

The first 500 days of data were used for training and the remaining 230 days for testing throughout the demand estimation phase. The neural network’s structure and programming were developed with the goal of validating the model as the data was being trained. As a result, 500 days of training were used to validate the neural network approach. The effectiveness of this model has been evaluated statistically. For this analysis, statistical variables including mean relative absolute error, mean absolute error, *R*2 value, and mean squared error were employed. The statistical error analysis of the neural network model is presented in Table 12. As can be observed, the model performs well in the training, validation, and testing phases.

**Table 12:** Parameters of neural network model statistical errors in three steps of training, validation and testing

**Step**	**MSE**	${{\varvec{R}}}^{2}$** constant**	**Absolute mean error**	**Mean relative absolute error**
Training	1.21	0.978	0.529	5.67
Validation	1.06	0.995	0.432	4.96
Testing	1.24	0.987	0.522	6.13

In the second step, the reordering point and the inventory of the suppliers and consumers were obtained over the course of a year. The simulation algorithm then used these order values to simulate and extract the period’s waste and shortages, which it then compared to the period’s actual waste and shortage. As the suggested model has been applied in six central databases and 54 hospitals, it must be highlighted that the data from the Mashhad central database was evaluated as a sample to evaluate the validity of the model. To begin with, a F test was used to determine that the variances of the two communities (the output of the simulation results and the data from the real system history) are not equal. An independent t-test was run with a 95% confidence level, knowing that the variances on the real sample data and the simulation model were not equal despite the assumption that the mean of both populations was equal. The results are shown in Tables 13 and 14, and they show that the value of p is acceptable and that the null hypothesis was confirmed. It indicated that the two populations’ means were equal. The findings indicate that there are no significant differences between the simulated results and the real data. As a consequence, it is safe to depend on the results of behavior simulations for bases and hospitals.

**Table 13:** Statistical results of mean return (expiration waste) of Mashhad Central Hospital (as the sample)

	**Real data**	**Simulation results**
**Monthly average**	751.07	752.14
**Standard deviation**	17.7	14.3
**n**	12	12
***P*-value**	0.967

**Table 14:** Statistical results of the average shortage of hospitals in mashhad

	**Real data**	**Simulation results**
**Monthly average**	55.67	58.2
**Standard deviation**	1.82	0.95
**n**	12	12
***P*-value**	0.987	

The multi-objective planning model is validated in the third stage by solving ten different numerical samples with conditions that are comparable to the problem hypotheses; the results of this step demonstrate the model’s feasibility.

## Discussion

The following questions were addressed in this study. What is the optimal model for managing blood logistics with the potential of returning goods in the three-level blood transfusion network? One may argue that the needs of customers and suppliers within the network should be taken into account in order to manage blood inventories as efficiently as possible. Due to the lack of a documented scientific system, it was determined from the facts at hand that consumer request information was unreliable. Therefore, the target inventory amount and reorder point of each supplier and consumer were determined by running the simulation algorithm and it was used as the basis of their inventory control policy. First, the real demand of consumers (hospitals) was estimated with the aid of neural network technique. The implementation of this technology has significantly decreased blood returns due to expiry and successfully avoids potential shortages.Finally, by using multi-objective planning, it is possible to decide which consumer demands should be given priority by each provider and how best to satisfy the customers. Repeating the implementation of this model has significantly reduced wastes and shortages. On the other hand, central bases are better able to reliably implement their inventory management policies since they have had the necessary supervision on the behavior of their consumers. In response to the question regarding the optimal level of blood stock in central blood transfusion bases and hospitals covered by the study. It can be said that if the central bases and hospitals behave according to the output of the reusable simulation algorithm that is communicated by the RBCs at the beginning of each year, they can show optimal behavior in the field of inventory management, leading to a reduction in the amount of waste and shortages. As a result, the bases have the best possible control over their inventory as well as the inventory behavior of their consumers, monitor and trust the incoming demand information, and work to meet all of their customers’ demands. The output of this algorithm indicates the amount of the target inventory that minimizes the amount of waste and shortages. In addition, by determining the reorder point for hospitals, a formal ordering policy will be made for them, and their inventory management will also have a scientific structure, and the amount of shortages, wastes, and returns of each hospital will be significantly reduced. Finally, each central base may augment the stock by donors if it lowers due to requests for blood donations because it knows how much of its target stock it has. What is the workload of the blood distribution devices in response to the proper and optimal routes of blood transfusion to the examined consumers? It can be argued that if the target bases distribute the blood needed by the consumers in accordance with the designed routing program, they will reduce the cost of transportation in addition to the speed with which the consumers may access their needs.

In general, it can be said that the implementation of the design model has outcomes such as reducing the time of blood transfer from central bases (suppliers) to their consumers, increasing the speed of blood delivery to consumers, increasing the average blood stock in central bases in order to prevent shortages and increase the ability to fulfill The needs of consumers in emergency situations, reducing the accumulation of distribution machines for consumers in the location of central bases, the method for requesting, consuming, and storing blood in the region with better control over the central bases on the performance of their consumers in the amount of inventory have been investigated.

The use of this model in a specific region eliminates the need to create separate models for each base in the region, distinguishing it from other models proposed in previous studies. Thus, using the current algorithm, it is possible to update events and develop current and desired behaviors. It should be mentioned that the implemented model had limitations, the existence of which caused the weakness of the implemented model. Among these limitations, the following can be mentioned: In the designed model, the emphasis is on the normal and routine conditions of consumers and suppliers, and critical and special cases are not considered. Also, the desired model can be implemented for networks that have at least four central bases. Finally, this study has been conducted for the four main blood groups and due to the complexity and lack of sufficient information in the study center, the eight main groups (${O}^{+}{, O}^{-}{AB}^{+}, { AB}^{-}, {A}^{-}{, A}^{+}$) is not included. Among the other limitations of the research that led to the simplification of the model, the following can be mentioned: In this research, only the model designed for one type of blood product (red blood cells) which has the highest request was examined and studied and other blood products are not considered. Also, one of the types of waste in the studied network was laboratory waste, the reasons of which can include rupture of the blood bag, malfunction of the device, and other such cases, which were not investigated in this study.

The blood transfusion organization should compile the inventory and distribution policy guidelines and communicate them to the central bases in order to implement the model designed in order to have the optimal impact on the inventory and distribution system. Additionally, each central base has to employ a permanent representative to investigate the cause of consumer waste.

## Suggestions for future research

By examining the research findings, suggestions can be made for future studies.

1. Forecasting specific crises and including them in the model can be considered in future research.
2. It is suggested to implement the desired model for other blood products.
3. Using the proposed model, while considering any possible requests from existing hospitals in the region.
4. Using the proposed model in other regions in the country’s blood transfusion organization and providing a documented ordering and inventory system to actors in each region.
5. In the proposed research model, in order to make the model closer to reality, eight main blood groups should be included in the model.
6. Entering all types of waste in the model, including laboratory waste, transportation waste, and such things can have a significant impact on the output of the proposed model.
7. Fuzzy approach can be used to increase the accuracy of data and reduce subjective judgment error in the implementation of routing model.

## Data Availability

The data that support the findings of this study are available from the corresponding author upon reasonable request.
